# The incidence of early post-operative complications following uvulopalatopharyngoplasty: identification of predictive risk factors

**DOI:** 10.1186/1916-0216-42-15

**Published:** 2013-02-06

**Authors:** Thileeban Kandasamy, Erin D Wright, John Fuller, Brian W Rotenberg

**Affiliations:** 1 Department of Otolaryngology – Head & Neck Surgery, University of Western Ontario, London, ON, Canada; 2 Department of Surgery, Division of Otolaryngology, University of Alberta, Edmonton, AB, Canada; 3 Department of Anesthesia and Perioperative Medicine, University of Western Ontario, London, ON, Canada; 4 St. Joseph’s Healthcare Centre, 268 Grosvenor St., London, ON, N6A 4V2, Canada

## Abstract

**Objective:**

Characterize complications following uvulopalatopharyngoplasty (UPPP) for obstructive sleep apnea.

**Study design:**

Retrospective chart review.

**Subjects and methods:**

Charts of patients undergoing UPPP at an academic teaching hospital from 1999 to 2005 were reviewed.

**Results:**

345 consecutive patients (248 inpatients; 97 outpatients) were studied. The most common post-operative complication in the entire study was oxyhemoglobin desaturation (12.8%). Three patients suffered major complications (airway obstruction, pulmonary edema, arrhythmia). Regarding complications limited to the post-anaesthetic care unit alone, only 8.2% of patients had oxyhemoglobin desaturation after discontinuation of oxygen supplementation. Inpatients requiring supplemental oxygen on the ward had significantly higher mean AHI (37.4 vs. 31.4; p=0.05) and BMI (32.3 kg/m^2^ vs. 28.9 kg/m^2^; p=0.004) than those who did not. Those inpatients who were obese (BMI > 30 kg/m^2^) with an AHI≥22 were associated with an increased risk of requiring oxygen on the ward (odds ratio = 3.48, 95% CI = 1.56 – 7.78).

**Conclusion:**

The incidence of post-UPPP complications is much lower than the literature has historically suggested. Selected patients should be able to safely undergo outpatient UPPP. Patients with higher AHI, higher BMI, or multiple comorbidities are at higher risk for postoperative complications and are most appropriate for overnight monitoring.

## Introduction

Obstructive sleep apnea (OSA) affects 9% of males and 3% of females between the ages of 30 and 60 in the United States [1]. Untreated OSA can result in daytime hypersomnolence, hypertension, and in severe cases, cardiopulmonary failure, motor vehicle accidents, and death. Uvulopalatopharyngoplasty (UPPP) is one of the most commonly performed surgical treatments for OSA in adults, and can be combined with nasal or tongue-base procedures to give the patient a true multi-level correction. Early studies regarding UPPP outcomes demonstrated a high rate of peri- and post-operative morbidity and mortality secondary to failed intubations, arrhythmias secondary to severe apnea, hemorrhage, or upper airway obstruction, and therefore recommended post-operative monitoring in an intensive care setting [2,3]. However, as UPPP has evolved it has become far safer and less morbid. Unlike early cases, tracheotomies are now rarely done in conjunction with UPPPs. Further several studies have shown that overnight monitoring in a step-down unit is a safe alternative to intensive care unit (ICU) monitoring [4-6]. As a result, most institutions currently monitor patients for 24 hours overnight in a step-down unit prior to discharge, and do not routine admit patients to the ICU.

More recently, studies have argued that in selected patients UPPP can be performed as an outpatient surgery [6-10]. These studies have consistently shown that complications rates are low, that more severe complications tend to occur while still in recovery room, and no postoperative mortality. However, these studies consisted of a limited number of patients and highly variable length of postoperative follow-up. Given the low incidence of serious complications, it is difficult to estimate complication rates accurately with small studies. Also, respiratory complications may be underestimated as in most studies post-operative oxygen supplementation was routinely given to patients - this was not independently reviewed in previous studies. In addition, direct comparisons of inpatient versus outpatient UPPP procedures is lacking in the literature.

We, therefore, performed a 5-year retrospective review of all adults undergoing inpatient and outpatient UPPP to (1) assess the complication rates and compare with those previously published in the literature, (2) compare complication rates between outpatient and inpatient UPPP, (3), identify preoperative characteristics associated with the development of later complications, (4) and to present recommendations for the postoperative management of UPPP patients.

## Methods

A retrospective review was performed of all patients undergoing UPPP (with or without septoplasty) between 1999 and 2005 at St. Joseph’s Health Care in London, Ontario, Canada. The research ethics board at the University of Western Ontario approved the study. A specially trained medical student, using direct entry to an electronic database, performed data abstraction. Abstraction of the first 20 patients was supervised by two of the senior authors to help ensure accuracy of the data. Preoperative polysomnography records, preoperative clinic notes, anesthesia records, post-anesthesia care unit (PACU) records, and ward records were reviewed. From these sources, apnea-hypopnea index (AHI), patent demographics, medical history, surgical procedure, ease of intubation, perioperative inhalation agents, muscle relaxants, and narcotics, postoperative narcotic use, oxyhemoglobin saturation, blood pressure, time of surgery and discharge, and all complications were recorded. All available co-morbidity data were obtained and divided into hypertension, hypercholesterolemia, cardiac, diabetes, and other. Patients with severe OSA-related comorbidities (such as cor-pumonale) were not included in the study, as their mandatorily higher level of post-operative care would have biased the results.

As over the study time period 10 different surgeon’s records were assessed to capture the largest number of patients undergoing UPPP, we allowed cases to be recorded only if the UPPP was a standard Fujita type UPPP (with or without tonsillectomy) performed with cautery. Laser-assisted cases, or cases done without cautery, being few and far between, were excluded. Only cases undergoing UPPP +/− nasal surgery were included in the study; patients undergoing any type of tongue base surgery had their records excluded.

We defined “early post-operative complication” as any complication occurring within the first two weeks after surgery. Data was not collected for problems after that time point. A surgical complication was intentionally very broadly defined as any untoward event occurring during anesthesia, surgery, or within the postoperative stay in hospital that may or may not have required active medical intervention. Such a broad definition was used so as to be able to capture all perioperative events. Complications were obtained from progress notes, nurse notes, discharge forms, surgeons’ clinic notes and hospital records.

Oxyhemoglobin desaturation was defined as oxyhemoglobin saturation less than 88% while the patient was asleep or awake. Of note, patients did not routinely receive post-operative CPAP therapy. Inpatients were monitored in the PACU for 2–3 hours and then moved to the inpatient ward. Outpatients were monitored in the PACU for 2–3 hours and then moved to the day surgery ward for 2–3 hours prior to discharge from hospital.

Comparisons of means between groups were done using unpaired Student’s t-test for variables with Gaussian distribution, and Mann–Whitney for non-Gaussian variables (AHI) based on the Shapiro-Wilks’ test. The presence or absence of risk factors between groups was compared by χ^2^ analysis. To determine the accuracy of parameters in predicting outcomes, odds ratios were calculated using SPSS (SPSS statistical package version 14.0; SPSS, Inc., Chicago, IL). Due to the remote nature and level of documentation available for some of the surgeons or cases, a subgroup analysis by surgeon (which would have been ideal) could not be performed. Thus the study sample was only analyzed as a whole.

## Results

### Demographics

A total of 345 patients treated by 10 different surgeons met the study criteria. Pre-operative AHI values were not available for 72 patients (20.9%) and therefore these patients’ data were not included in any analysis involving AHI values. Thirty-four (47.2%) of these patients without preoperative AHI values were inpatients while the remaining 38 (52.8%) were outpatients. The patient population consisted of 295 males and 50 females with a mean age of 35.7 (range, 15 to 59; standard deviation [SD] = 9.1), a mean apnea hypopnea index (AHI) of 32.8 (range, 2 to 128; SD = 24.3), and a mean body mass index (BMI) of 30.4 kg/m^2^ (range, 18.7 to 56.6 kg/m^2^; SD = 8.4). A total of 248 of these patients were monitored overnight in the ward while 97 patients were discharged on the same day of their surgery. Outpatients had a lower mean age (outpatients: 33.2; inpatients: 36.8; p=0.001; 95% CI = 4.8 to 18.7) and had a significantly lower AHI (outpatients: 23.6; inpatients: 35.3; p=0.001; 95% CI = 1.5 to 5.8). No significant differences were found between groups with respect to the presence of co-morbidities (Table 1). Surgical procedures that were performed simultaneously with UPPPs were septoplasty, inferior turbinate reduction, and polypectomy. The number of these procedures was not significantly different in the inpatient and outpatient groups. In addition, no statistical difference was found between complication and non-complication groups when all surgical procedures were analyzed as independent risk factors (Table 2).

**Table 1:** Comparison of outpatient and inpatient UPPP

	**Outpatient (n=97)**		**Inpatient (n=248)**		**p**
	**Mean**	**SD**	**Mean**	**SD**	
Age	33.2	7.6	36.8	9.5	0.001
BMI (kg/m^2^)	29.1	4.4	30.1	9.5	0.07
AHI	23.6	15.8	35.32	25.6	0.001
Co-morbidities (%)	45.4		50.4		0.4
Complications					
Total (%)	21.6	-	33.9	-	0.027
Respiratory (%)	5.2	-	15.7	-	0.008
Hemorrhage (%)	8.2	-	13.3	-	0.163
Hypertension (%)	3.2	-	5.1	-	0.48

**Table 2:** Surgical procedures performed in addition to UPPP

	**No complications**		**Complications**		**p**
	**N**	**%**	**N**	**%**	
Tonsillectomy	94	38.8	42	40.8	0.413
Septoplasty	17	7.0	8	7.8	0.483
ITR*	6	2.5	2	1.9	0.555
Polypectomy	1	0.004	1	0.01	0.213

*Inferior Turbinate Reduction.

### Complications

No deaths occurred within the two-week postoperative period during which data was collected for the study. The incidence of all postoperative complications (major and minor) was 28.1% (n=97). The total complication rate in the inpatient group (31.3%) was significantly higher (p=0.04) than that of the outpatient group (20.6%). When only comparing PACU complications, no significant difference (p=0.09) was found for total complications in the inpatient (30.3%) and outpatient (20.3%) groups.

Major complications occurred in 3 inpatients and all occurred within 2 hours post-surgery. One patient suffered laryngospasm during extubation and required bag-mask ventilation. This patient subsequently developed pulmonary edema that was treated with supplemental oxygen and diuresis. Two other patients developed sinus bradycardia and were monitored, but it resolved spontaneously without treatment. None of these complications were thought to be directly related to the patient’s OSA.

The most common minor complication after surgery was oxyhemoglobin desaturation at an incidence of 12.8% (n=44). The desaturation rate of the inpatient group (15.7%) was significantly higher (p=0.008) than that of the outpatient group (5.2%). When comparing oxyhemoglobin desaturation rates only occurring in the PACU (and not on the ward), there was no significant difference in the inpatient (6.0%) and outpatient (4.1%) groups (p=0.5). The incidence of oxyhemoglobin desaturations was not related to the incidence of co-morbidities in these categories. The majority of these complications occurred when oxygen supplementation was discontinued to determine oxyhemoglobin saturation on room air. All oxyhemoglobin desaturations in the outpatient group occurred in the PACU. It was also noted that of the 80 inpatients who were able to maintain oxyhemoglobin saturation >88% on room air in the PACU, only 8.75% subsequently suffered a desaturation on the ward. These oxyhemoglobin desaturation complications occurred during sleep and were treated with supplemental oxygen.

Bleeding of any sort (nasal or pharyngeal) occurred in 15 (4.3%) patients. The rates in the inpatient group (5.1%) did not differ significantly (p=0.48) than that of the outpatient group (3.2%). All bleeding complications in the outpatient groups occurred at least 3 days after discharge and were treated with cautery, packing, or an admission to the hospital for monitoring. Seven of the twelve inpatients with hemorrhagic complications occurred after discharge from hospital (>24 hours post-surgery). Three inpatients required operative intervention to control post-tonsillectomy bleeding.

Seven patients (2%) returned to the hospital >24 h post-discharge for uncontrolled pain, all of which were treated uneventfully with narcotics for pain control, and none suffered any adverse respiratory outcome from this. Other complications included 2 cases of severe nausea post-discharge requiring hospital admission for intravenous fluids, and 3 cases of post-discharge infection requiring admission (2 patients) or oral antibiotics (1 patient).

### Identifying risk factors

A comparison of patient groups with and without complications showed no statistical differences with respect to age, sex, BMI, AHI, or the presence of co-morbidities. Statistically significant differences were noted, however, between those patients who suffered post-PACU complications compared to those who did not. Patients with post-PACU complications had a higher incidence of any co-morbid illness (p=0.03) and multiple co-morbidities (p=0.02) (Table 3). No single co-morbidity was independently significant on its own.

**Table 3:** Post-PACU complications

	**No post-PACU complications (n=289)**		**Post-PACU complications (n=56)**		**p**
	**Mean**	**SD**	**Mean**	**SD**	
Age	35.4	9.3	37.1	7.7	0.22
BMI (kg/m^2^)	30.2	8.8	31.6	5.7	0.26
AHI	33.0	24.5	35.8	23.9	0.51
Co-morbidity (%)	46.4	-	62.5	-	**0.03**
Multiple co-morbidities	8.0	-	17.9	-	**0.02**

To identify risk factors associated with the development of oxyhemoglobin desaturation complications, a subgroup analysis of all inpatients was performed. No significant differences were found between inpatients with and without oxyhemoglobin desaturation complications with respect to all obtained variables. Given that respiratory complication rates were skewed by post-operative oxygen supplementation, a comparison was made of inpatients that were successfully taken off of oxygen supplementation in the PACU without further need for oxygen (n=75) with those who required oxygen on the ward (n=133). When compared to inpatients that required oxygen, those who were taken off of supplemental oxygen had a significantly lower mean AHI (31.44 ± 3.4 vs. 37.39 ± 2.5; p=0.05) and significantly lower mean BMI (28.9 ± 0.5 kg/m^2^ vs. 32.3 ± 1.0 kg/m^2^; p=0.004). To further investigate these risk factors, odds ratios (ORs) were calculated. Patient who had an AHI ≥ 22 were more likely to require oxygen in the PACU (OR = 2.21, 95% CI = 1.166 - 4.188), although the duration of oxygen usage was not obtainable from patient records. Patients who were obese (BMI ≥ 30) were also more likely to require oxygen in the PACU (OR = 2.70, 95% CI = 1.48 - 4.91). Interestingly, patients who had both an AHI ≥ 22 and a BMI ≥ 30 were even more likely to require oxygen in the PACU (OR = 3.48, 95% CI = 1.56 – 7.78).

## Discussion

This study represents the largest multi-surgeon series in the literature of patients undergoing UPPP with or without additional procedures as both outpatient and inpatient surgery individually and combined. Ninety-seven patients (28.1%) were discharged on the day of the surgery. Only three patients suffered serious complications, all occurring within two hours of surgery, that could be directly associated to the underlying OSA.

Fairbanks et al [2]. describe 12 anecdotal cases of post-operative mortality secondary to airway obstruction. However, no information is available regarding patient’s preoperative health, OSA severity, or postoperative course in that study. Haavisto & Juonpaa [11] describe one case of death immediately after extubation. Kezirian et al [12]. describe 7 deaths in 3130 patients following UPPP, but the causes of death are not described. No other studies report post-operative death secondary to airway complications. Recent studies show no postoperative mortality [5,7,8,13-15]. This is likely due to increased awareness of risk factors, decreased narcotic use, and perioperative steroid use.

The incidence of all respiratory complications (including laryngospasm and pulmonary edema) in our series was 13.3%; when examining only uncomplicated oxygen desaturations, the incidence was lower at 12.8%. Respiratory complication rates in the literature range from 1.4% [6] to 15.3% [14]. Some authors only include serious respiratory complications such as pneumonia, tracheostomy, or pulmonary edema, and do not consider a simple oxygen desaturation to be a complication. In addition, many centres in previous studies provide their OSA population oxygen supplementation until discharge from hospital as routine care. These factors may explain the variability in the respiratory complication rates stated in previous studies. In our study, all respiratory complications, with the exception of one case of laryngospasm, were that of decreased oxyhemoglobin saturation and were treated with oxygen supplementation. It is questionable however if simple desaturations should actually be considered complications in this population. Patients with OSA have generally been desaturating nightly for years prior to surgery as part of their OSA, and thus it may be invalid to consider post-operative desaturations as causally related to the UPPP as opposed to the underlying disease status. It is also questionable whether routine oxygen administration should be given after OSA surgery to treat desaturations. These topics are significantly understudied in the OSA literature, and merit further investigation.

Comparison of inpatient and outpatient groups showed that outpatients were significantly younger and had a significantly lower mean AHI. This likely represents a selection bias as surgeons may have preferentially selected younger patients with mild OSA for outpatient UPPP. The incidence of total complications and oxyhemoglobin desaturations in the PACU alone were not significantly different between inpatient and outpatient groups. This result suggests that complications in the PACU may not have influenced surgeon’s decisions to discharge patients.

Our results suggest that the most significant risk factor for the development of post-PACU respiratory complications is the presence of single or multiple co-morbidities. Previous studies describe AHI [3,14-16], preoperative LSAT (lowest oxyhemoglobin saturation) [13,14,16], BMI [13], or no factors [6,8] related to the incidence of postoperative respiratory complications. This discrepancy likely represents the inaccuracy in determining the rate of respiratory complications due to oxygen supplementation post-operatively. To correct for this factor, we compared patients undergoing UPPP with or without tonsillectomy that permanently stopped oxygen supplementation in the PACU while maintaining an oxyhemoglobin saturation > 88% with those requiring oxygen in the ward. We found that both BMI and AHI were significantly higher in patients requiring supplemental oxygen in the ward. Odds ratio calculations revealed that obese and morbidly obese patients (BMI ≥ 30) who had an AHI ≥ 22 were at significant risk of requiring oxygen supplementation on the ward and may warrant an overnight stay. Our results, therefore, suggests that these AHI and BMI values can be useful for identifying patients who can undergo safe outpatient UPPP surgery. It should be noted that it is likely that many patients received oxygen supplementation on the ward unnecessarily as a trial of oxygen discontinuation was not attempted in the PACU in all patients. This may bias our results.

The decision to discharge postoperative patients varies across surgical centres. Chung et al [17]. devised the post-anesthesia discharge scoring system (PADS) for providing a uniform assessment of all ambulatory surgical patients based on vital signs, pain, ambulation, nausea / vomiting, and bleeding. Specifically, vital signs within 20% of preoperative baseline are generally considered as adequate prior to discharge. All previous studies looking at postoperative sleep studies show no significant change in post-operative AHI, LSAT (lowest oxyhemoglobin saturation), or both in the immediate post-operative setting compared to preoperative levels [8,18,19]. In our study, 91.3% of all inpatients who were successfully taken off oxygen supplementation in the PACU did not require further oxygen treatment overnight. This data suggests that the risk of postoperative oxyhemoglobin desaturation in OSA patients is not significantly elevated compared to their preoperative baseline.

There are a few limitations to our study. Being a retrospective study, there is potential for a selection bias. However, we strictly adhered to our inclusion and exclusion criteria. As in previous studies, our respiratory complication rates may have been skewed by postoperative oxygen supplementation. We tried to account for this by recording oxygen use postoperatively and utilizing this data in our analysis. A key variable of lowest oxygen saturation level could not be identified in many of the records, therefore limiting the conclusions that can be drawn from this review. In addition, many patients did not have available preoperative AHI values. It is unclear whether this may have introduced a surgical selection bias. The level of CPAP adherence prior to surgery was not available for many records, especially the remote ones; CPAP adherence pre-operatively can in some cases affect respiratory functioning after surgery, hence this variable could not be accounted for in our study. However, it is known that the overall rate of CPAP adherence is low especially in a study population of patients undergoing surgery, hence we do not feel the lack of this variable will significantly affect the overall findings of this study. Finally, the decision to perform outpatient UPPP patients was generally based on surgeon’s preference. Although patients were required to maintain oxyhemoglobin saturation > 88% with pain and nausea controlled on oral medication, some surgeons may have elected to keep patients overnight despite patient factors. This may bias comparisons of outpatient and inpatient UPPP.

## Conclusion

Our study suggests that the rate of complications following isolated UPPP is low. All serious complications were detected within 2 to 3 hours of surgery. All bleeding complications following outpatient UPPP occurred at least 3 days post discharge from hospital and, therefore, likely would not have been prevented by 24-hour postoperative monitoring. Our study is the first to demonstrate that once patients are taken off oxygen supplementation in the PACU, they are likely not to require further oxygen supplementation. In addition, our study is the first to identify characteristics (AHI and BMI) associated with the need for oxygen therapy following UPPP.

We suggest that in many cases isolated UPPP can safely be performed as an outpatient procedure. Patients can be discharged from hospital once they are able to maintain adequate oxyhemoglobin saturation on room air in the PACU. Patients with BMI ≥ 30 and/or AHI ≥ 22 are more likely to require oxygen in the post-PACU setting and should be monitored overnight. Future studies can prospectively evaluate whether patients with these risk factors should be monitored in hospital overnight.

In the modern era of multilevel surgery it is recognized that many OSA patients undergoing surgery received not only a UPPP but often pharyngeal or tongue base procedures as well. Our study was limited to patients undergoing UPPP alone, hence we make no comment about complications in the setting of more advanced or multilevel surgery.

## Competing interests

All authors declare that they have no competing interests.

## Authors’ contributions

TK: Carried out study design, data collection, data analysis, and wrote portions of the manuscript. EW: Carried out study design and reviewed manuscript. JF: Carried out study design and reviewed manuscript. BR: Performed data analysis, and wrote portions of manuscript. All authors read and approved the final manuscript.

## Authors’ information

**Presented at** American Academy of Otolaryngology – Head and Neck Surgery Annual Meeting. Washington, DC. September 16–19, 2007.

## References

[B1] Young T Skatrud J Peppard PE Risk factors for obstructive sleep apnea in adults JAMA 2004 291 2013 10.1001/jama.291.16.2013 15113821

[B2] Fairbank DN Uvulopalatopharyngoplasty complications and avoidance strategies Otolaryngol Head Neck Surg 1990 102 239 245 2108411 10.1177/019459989010200306

[B3] Esclamado RM Glen MG McCulloch TM Perioperative complications and risk factors in the surgical treatment of obstructive sleep apnea syndrome Laryngoscope 1989 99 1125 1129 2530406 10.1288/00005537-198911000-00004

[B4] Terris DJ Fincher EF Hanasono MM Conservation of resources: indications for intensive care monitoring after upper airway surgery on patients with obstructive sleep apnea Laryngoscope 1998 108 784 788 10.1097/00005537-199806000-00002 9628489

[B5] Gessler EM Bondy PC Respiratory complications following tonsillectomy/UPPP: is step-down monitoring necessary? Ear Nose Throat J 2003 82 628 632 14503103

[B6] Mickelson SA Hakim I Is postoperative intensive care monitoring necessary after uvulopalatopharyngoplasty? Otolaryngol Head Neck Surg 1998 119 352 356 10.1016/S0194-5998(98)70077-4 9781989

[B7] Spiegel JH Tejas RH Overnight hospital stay is not always necessary after uvulopalatopharyngoplasty Laryngoscope 2005 115 167 171 10.1097/01.mlg.0000150703.36075.9c 15630388

[B8] Hathaway B Johnson JT Safety of uvulopalatopharyngoplasty as outpatient surgery Otolaryngol Head Neck Surg 1996 134 542 544 16564369 10.1016/j.otohns.2005.12.004

[B9] Rotenberg B Theriault J Pang K Is overnight monitoring required for adult patients undergoing surgery for obstructive sleep apnea? Laryngoscope 2011 121 4 692 693 10.1002/lary.21729 21433013

[B10] Rotenberg B Hu A Fuller J Bureau Y Arra I Sen M The early postoperative course of surgical sleep apnea patients Laryngoscope 2010 120 5 1063 1068 20222023 10.1002/lary.20889

[B11] Haavisto L Suonpaa J Complications of uvulopalatopharyngoplasty Clin Otolaryngol 1994 19 243 247 10.1111/j.1365-2273.1994.tb01224.x 7923849

[B12] Kezirian EJ Weaver EM Yueh B Incidence of serious complication after uvulopalatopharyngoplasty Laryngoscope 2004 114 240 243 10.1097/00005537-200403000-00012 15091217

[B13] Kieff DA Busaba NY Same-day discharge for selected patients undergoing combind nasal and palatal surgery for obstructive sleep apnea Ann Otol Rhinol Laryngol 2004 113 128 131 14994768 10.1177/000348940411300209

[B14] Kim JA Lee JJ Jung HH Predictive factors of immediate postoperative complications after uvulopalatopharyngoplasty Laryngoscope 2005 115 1837 1840 10.1097/01.mlg.0000173199.57456.2b 16222206

[B15] Pang KP Identifying patients who need close monitoring during and after upper airway surgery for obstructive sleep apnea J Laryngol Otol 2006 120 655 660 16740205 10.1017/S0022215106001617

[B16] Riley RW Powell NB Guilleminault C Obstructive sleep apnea surgery: risk management and complications Otolaryngol Head Neck Surg 1997 117 648 652 10.1016/S0194-5998(97)70047-0 9419093

[B17] Chung F Recovery pattern and home-readiness after ambulatory surgery Anesth Analg 1995 80 896 902 7726431 10.1097/00000539-199505000-00008

[B18] Burgess LPA Derderian SS Morin GV Post-operative risk following uvulopalatopharyngoplasty for obstructive sleep apnea Otolaryngol HeadNeck Surg 1992 106 81 86 10.1177/019459989210600132 1734375

[B19] Sanders MH Johnson JT Keller FA The acute effects of uvulopalatopharyngoplasty on breathing during sleep in sleep apnea patients Sleep 1988 11 1 75 89 3363273 10.1093/sleep/11.1.75

